# Kynurenine metabolic balance is disrupted in the hippocampus following peripheral lipopolysaccharide challenge

**DOI:** 10.1186/s12974-016-0590-y

**Published:** 2016-05-27

**Authors:** Jennifer M. Parrott, Laney Redus, Jason C. O’Connor

**Affiliations:** Department of Pharmacology, School of Medicine, Center for Biomedical Neuroscience, University of Texas Health Science Center at San Antonio, 422D Medical Building MC-7764, 7703 Floyd Curl Drive, San Antonio, TX 78229-3900 USA; Department of Pharmacology, School of Medicine, University of Texas Health Science Center at San Antonio, 418D Medical Building MC-7764, 7703 Floyd Curl Drive, San Antonio, TX 78229-3900 USA; Department of Pharmacology, School of Medicine, Center for Biomedical Neuroscience and Mood Disorders Translational Research Core, University of Texas Health Science Center at San Antonio, 216B Medical Building MC-7764, 7703 Floyd Curl Drive, San Antonio, TX 78229-3900 USA; Audie L. Murphy Memorial VA Hospital, South Texas Veterans Health System, 7400 Merton Minter, San Antonio, Texas 78229-4404 USA

**Keywords:** Neuroinflammation, Kynurenine, Pro-inflammatory cytokines, Brain regions, Microglia, Indoleamine 2,3-dioxygenase, Kynurenine 3-monooxygenase, Hippocampus

## Abstract

**Background:**

Inflammation increases the risk of developing depression-related symptoms, and tryptophan metabolism is an important mediator of these behavior changes. Peripheral immune activation results in central up-regulation of pro-inflammatory cytokine expression, microglia activation, and the production of neurotoxic kynurenine metabolites. The neuroinflammatory and kynurenine metabolic response to peripheral immune activation has been largely characterized at the whole brain level. It is unknown if this metabolic response exhibits regional specificity even though the unique indoleamine 2,3-dioxygenase (IDO)-dependent depressive-like behaviors are known to be controlled by discrete brain regions. Therefore, regional characterization of neuroinflammation and kynurenine metabolism might allow for better understanding of the potential mechanisms that mediate inflammation-associated behavior changes.

**Methods:**

Following peripheral immune challenge with lipopolysaccharide (LPS), brain tissue from behaviorally relevant regions was analyzed for changes in mRNA of neuroinflammatory targets and kynurenine pathway enzymes. The metabolic balance of the kynurenine pathway was also determined in the peripheral circulation and these brain regions.

**Results:**

Peripheral LPS treatment resulted in region-independent up-regulation of brain expression of pro-inflammatory cytokines and glial cellular markers indicative of a neuroinflammatory response. The expression of kynurenine pathway enzymes was also largely region-independent. While the kynurenine/tryptophan ratio was elevated significantly in both the plasma and in each brain regions evaluated, the balance of kynurenine metabolism was skewed toward production of neurotoxic metabolites in the hippocampus.

**Conclusions:**

The upstream neuroinflammatory processes, such as pro-inflammatory cytokine production, glial cell activation, and kynurenine production, may be similar throughout the brain. However, it appears that the balance of downstream kynurenine metabolism is a tightly regulated brain region-dependent process.

## Background

The behavioral consequences of inflammation and pro-inflammatory cytokines have been well described, both clinically and preclinically. Chronic diseases, often characterized by prolonged immune activation, are associated with an increased risk of comorbid depression diagnosis [1]. Approximately 50–80 % of patients receiving interferon-α (IFN-α) immunotherapy for hepatitis C or malignant melanoma develop depression-related symptoms during the course of treatment, including neurovegetative, affective, and/or cognitive impairment [2]. Administration of endotoxin or vaccines to healthy volunteers induces transient depression-associated symptoms that parallel the appearance of pro-inflammatory cytokines [3–6], whereas direct administration of pro-inflammatory cytokines in preclinical models recapitulates inflammation-associated depressive-like behaviors [7–10]. Pharmacological or genetic inhibition of pro-inflammatory cytokine action effectively attenuates many of the inflammation-induced depressive-like behaviors in rodents, establishing that pro-inflammatory cytokines are necessary to precipitate this behavioral response [11, 12]. Clearly, pro-inflammatory cytokines are important pathogenic mediators of depression-related behavior changes resulting from immune stimulation; however, the specific mechanism by which this occurs is not clearly defined.

**Fig. 1:**
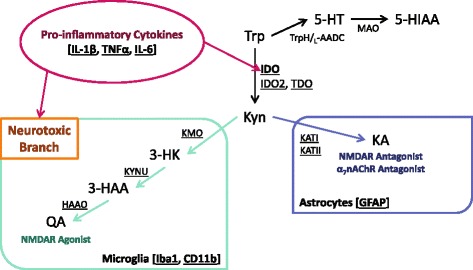
Kynurenine pathway of tryptophan metabolism. Tryptophan is metabolized to kynurenine by indoleamine 2,3-dioxygenase (*IDO*), IDO-like enzyme, *IDO2*, and tryptophan 2,3-dioxygenase (*TDO*). Tryptophan can also be metabolized by tryptophan hydroxylase (*TrpH*) and l-aromatic amino acid decarboxylase (_*L*_ *-AADC*) to serotonin (*5-HT*) which is further broken down by monoamine oxidase (*MAO*) to 5-hydroxyindoleacetic acid (*5-HIAA*). Kynurenine can be metabolized to kynurenic acid (*KA*) by one of four isoforms of kynurenine aminotransferase (KAT), of which *KATI* and *KATII* are most relevant to mammals. The production of KA by KATs mainly occurs in astrocytes, cells which predominantly express glial fibrillary acidic protein (*GFAP*). Alternately, kynurenine can be metabolized to 3-hydroxykynurenine (*3-HK*) by kynurenine 3-monooxygenase (*KMO*). 3-HK is metabolized to 3-hydroxyanthranilic acid (*3-HAA*) by kynureninase (*KYNU*) and 3-HAA to quinolinic acid (*QA*) by 3-hydroxyanthranilic acid dioxygenase (*HAAO*). This branch of the kynurenine pathway is compartmentalized in microglia, indicated by ionized calcium-binding adapter molecule 1 (*Iba1*) and *CD11b* expression. The elevation in expression of pro-inflammatory cytokines, such as interleukin-1β (*IL-1β*), tumor necrosis factor α (*TNFα*), and interleukin-6 (*IL-6*), during inflammatory conditions activates glial cells. Further, IDO expression is up-regulated and flux through the neurotoxic kynurenine metabolic branch increases. *Underlined* targets were assessed by real-time RT-PCR to determine changes in mRNA

Peripheral immune activation and subsequent production of pro-inflammatory cytokines communicate immune status to the brain via multiple routes, where the inflammatory signals are locally propagated by microglia, the resident immune cells [13]. While the initial neuroinflammatory response may be part of an adaptive beneficial process, excessive or chronic neuroinflammation has been associated with adverse neurological consequences [14, 15]. These consequences can include the disruption of neurotransmission, neurotrophin signaling, hypothalamic-pituitary-adrenal (HPA) axis activation, and alteration of metabolic pathways [13]. One metabolic pathway hypothesized to be important in mediating the effects of pro-inflammatory cytokines in the brain is the kynurenine pathway of tryptophan metabolism (Fig. 1), the major route of central tryptophan breakdown [16]. Pro-inflammatory cytokines up-regulate the expression and activity of indoleamine 2,3-dioxygenase (IDO, Fig. 1), the rate-limiting enzyme of the kynurenine pathway, increasing flux through the pathway [17–19]. During basal conditions, kynurenine is further metabolized to kynurenic acid (KA, Fig. 1), an *N*-methyl-d-aspartate receptor (NMDAR) antagonist and an α_7-_nicotinic acetylcholine receptor (α_7_nAChR) negative allosteric modulator [20, 21]. However, during neuroinflammatory conditions, the “neurotoxic” branch of kynurenine metabolism is up-regulated, which drives the synthesis of several neuroactive metabolites, including quinolinic acid (QA, Fig. 1), an NMDAR agonist and neurotoxic metabolite [20, 22, 23]. This shift in kynurenine pathway balance, increasing the production of neurotoxic QA relative to KA, that occurs during neuroinflammation, is hypothesized to underlie inflammation-associated depression-related behaviors [24]. Inhibition or genetic removal of IDO demonstrated the necessity of kynurenine metabolism to the development of inflammation-induced depressive-like behaviors in rodents [19, 25, 26]. Further, antagonism of the NMDAR with ketamine attenuated inflammation-induced anhedonia and behavioral despair, implicating activation of NMDAR by QA, as a mechanism that induces inflammation-associated depressive-like behaviors [23]. Interferon-α immunotherapy resulted in elevated serum kynurenine/tryptophan ratio, indicative of increased IDO activity, as well as increased cerebrospinal fluid (CSF) levels of kynurenine, KA, and QA [27, 28]. Only CSF QA was significantly positively associated with Montgomery-Asberg Depression Rating Scale (MADRS) scores in these patients [28]. However, to date, inflammation-induced changes in kynurenine metabolism have largely been measured in whole brain samples even though distinct, regionally specific neurocircuitry controls the various symptom dimensions of depression.

Depression symptomatology is heterogeneous throughout the patient population, complicating research efforts targeted at understanding the neurobiological substrates underlying the disease. Because of this, the National Institute of Mental Health (NIMH) established the Research Domain Criteria (RDoC, http://www.nimh.nih.gov/research-priorities/rdoc/index.shtml) as a construct for mental health research [29, 30]. Rather than defining patient populations by a disease diagnosis, the RDoC instead prompts investigators to focus on understanding the mechanism that underlies specific symptoms or behaviors. In healthy volunteers, endotoxin administration results in anhedonia, disruption in cognitive performance, anxiety, and mood disturbances [31, 32]. These endotoxin-associated symptoms have been associated with altered blood flow (imaged using functional magnetic resonance imaging (fMRI)) in the ventral striatum, amygdala, and dorsomedial prefrontal cortex among other regions [5, 33, 34]. Similar to human patients, lipopolysaccharide (LPS, endotoxin) challenge in mice results in similar behaviors (anhedonia, anxiety, behavioral despair, cognitive disruption) [19, 25, 26]. Determining the relevant regional neuroinflammatory response, particularly kynurenine metabolism, following peripheral immune activation with LPS will lead to a better understanding of the development of these behaviors.

While the region-specific expression of pro-inflammatory cytokines after peripheral immune challenge has been partially characterized [17, 35], the impact of peripheral LPS challenge on kynurenine pathway enzyme expression and subsequent metabolism has only been described at the whole brain level [23]. Here, we tested the hypothesis that up-regulation of kynurenine pathway enzymes and disruption of kynurenine metabolic balance occurs in a regionally distinct manner. Brain regions associated with depressive-like behaviors were collected following peripheral immune challenge with LPS in mice. The steady-state mRNA expression of markers of glial activation, pro-inflammatory cytokines, and kynurenine pathway enzymes were measured. Tryptophan and its metabolites were also measured in each brain region. The brain regions assessed included the hippocampus, the amygdala, and the ventral striatum (nucleus accumbens). Dysfunction in these brain regions has been reported in depressed patients and is hypothesized to be a critical pathogenic mechanism underlying distinct depression-associated behaviors [36]. Because the periphery is reported to provide much of the brain’s kynurenine [37], plasma metabolite levels were also measured. The data demonstrate that peripheral LPS treatment elevates the expression of pro-inflammatory cytokines and glial cell markers similarly in each region. IDO expression, but not IDO2 or tryptophan 2,3-dioxygenase (TDO), was significantly up-regulated after LPS treatment. Consistent with IDO up-regulation, the kynurenine/tryptophan ratio was elevated in each region; however, the 3-hydroxykynurenine (3-HK)/KA ratio, an indicator of a neurotoxic imbalance in kynurenine metabolism, was only increased in the hippocampus (dorsal and ventral). These data are the first that characterize the brain region-specific metabolic response of the kynurenine pathway following peripheral LPS challenge.

## Methods

### Animals

All animal care and use was conducted in accord with the Guide for the Care and Use of Laboratory Animals, eighth edition (NRC), and protocols were approved by the Institutional Animal Care and Use Committee at the University of Texas Health Science Center at San Antonio (UTHSCSA). Male C57BL/6J mice were obtained from Jackson Laboratory (Bar Harbor, ME; stock# 000664) and were used at ages between 8 and 12 weeks of age (adults). Prior to use, mice were group housed in standard shoebox cages, allowed *ad libitum* food and water access, and general health was monitored daily by veterinary technicians or research staff.

### Treatment

Lipopolysaccharide (Sigma, St. Louis, MO) isolated from *Escherichia coli* (L-3129, serotype 0127:B8) was prepared fresh on the morning of injections and was dissolved in sterile, endotoxin-free 0.9 % saline (vehicle) and injected intraperitoneally (i.p.) at a dose of 0.5 mg/kg.

### Tissue sample collection

At either 6 or 24 h following saline or LPS injections, mice were euthanized by carbon-dioxide asphyxiation. To measure mRNA expression of target genes, brain regions (amygdala, hippocampus, striatum) were grossly dissected (based on stereological coordinates in a mouse brain atlas [38]). Subsequent analysis of brain region metabolites was performed on microdissected brain regions harvested from serial 1-mm coronal brain sections using a brain matrix (Stoelting Co., Wood Dale, IL) (based on stereological coordinates in a mouse brain atlas [38]). Tissue was snap frozen in liquid nitrogen and stored at −80 °C until analyzed. At 24 h following injections, prior to perfusion, blood was also collected for separation of plasma, which was stored at −80 °C.

### RNA isolation and real-time RT-PCR

Brain tissues (hippocampus, amygdala, striatum) collected at 6 and 24 h post treatment (saline, LPS) were analyzed to determine relative steady-state mRNA expression using real-time RT-PCR. Isolation of RNA was carried out according manufacturer instructions using a PureLink® RNA Mini Kit (Life Technologies, Grand Island, NY), and cDNA was created using a high-capacity cDNA RT kit (Life Technologies) following manufacturer instructions. Real-time RT-PCR was performed over 40 cycles using a CFX384™ Real-Time PCR Detection System (Bio-Rad, Hercules, CA) and Taqman® Gene Expression Assays (Life Technologies): *Gapdh* (GAPDH, Mm99999915_g1), *Il1b* (IL-1β, Mm01336189_m1), *Tnf* (TNFα, Mm00443258_m1), *Il6* (IL-6, Mm00446190_m1), *Itgam* (CD11b, Mm00434455_m1), *Aif1* (Iba1, Mm00479862_g1), *Gfap* (GFAP, Mm01253033_m1), *Ido* (IDO, Mm00492586_m1), Ido2 (IDO2, Mm00524206_m1), Tdo2 (TDO, Mm00451266_m1), *Ccbl1* (KATI, Mm01327703_m1), *Aadat* (KATII, Mm00496169_m1), *Kmo* (KMO, Mm00505511_m1), *Kynu* (KYNU, Mm00551012_m1), and *Haao* (HAAO, Mm00517945_m1). Data are expressed as relative fold change (Target Δ_mRNA_) using the 2^−ΔΔCt^ calculation method and GAPDH as the housekeeping gene as previously described [12].

### Liquid chromatography/mass spectrometry

Microdissected brain regions (dorsal and ventral hippocampus, central amygdala, nucleus accumbens) and plasma were prepared for liquid chromatography/mass spectrometry (LC/MS) and analyzed for kynurenine metabolites as previously described [23]. Briefly, thawed plasma samples were diluted five times with 0.2 % acetic acid and 1 mM internal standards, transferred to Amicon Ultra filters (Millipore, Billerica, MA) and centrifuged at 13,500×*g* for 1 h at 4 °C. Frozen brain regions were diluted 30 times with 0.2 % acetic acid and 1 mM internal standards and then homogenized at 4 °C using a Bead Ruptor 24 Homogenizer (Omni International, Kennesaw, GA) with 1.4-mm zirconium ceramic oxide beads (Omni International) and settings of (pulse duration 45 s, pulse number 2, rest interval 15 s). The supernatant was filtered in a centrifuge as the plasma. Following preparation, samples were analyzed on a Q Exactive mass spectrometer (Thermo Fisher Scientific, Waltham, MA) with on-line separation by a Dionex UltiMate 3000 HPLC system (Thermo Fisher Scientific), and the data collected was analyzed using Xcalibur 2.2 software (Thermo Fisher Scientific) in the Mass Spectrometry Core Facility at the University of Texas Health Science Center at San Antonio. Metabolite ratios were calculated, as an in vivo estimation of IDO activity, kynurenine metabolic balance, and serotonin turnover, from raw data that are part of a separate study (Parrott JM, Redus L, Morales J, Xiaoli G, O’Connor JC: Neurotoxic kynurenine metabolism is increased in the dorsal hippocampus and drives distinct depressive behaviors during inflammation, Submitted). These values were used to determine the following ratios: kynurenine/tryptophan, 3-HK/KA, and 5-HIAA/5-HT. 3-Hydroxyanthranilic acid (3-HAA) and QA were not reliably detected in brain region microdissected samples due to limited sample size.

### Statistical analysis

Data were analyzed using SigmaPlot 12.0 software (Systat Software Inc., San Jose, CA) and are presented as group means + standard error of the mean (SEM). Following a single-pass Chauvenet’s test for outliers as previously described [26], data analysis was conducted using either a *t* test or a one-way analysis of variance (ANOVA). Significant main effects identified by the ANOVA were further analyzed with the Holm-Sidak method for pairwise multiple comparisons post hoc test to identify between-group differences. Significant treatment effects (*p* < 0.05, compared to saline) are denoted as such (*), and trends (*p* < 0.1–0.05) are denoted as such (^#^) (^#^*p* < 0.1–0.05; *p < 0.05–0.01; **p < 0.01–0.001; ***p < 0.001).

## Results

### Peripheral lipopolysaccharide induces time-dependent central pro-inflammatory cytokine expression

**Fig. 2:**
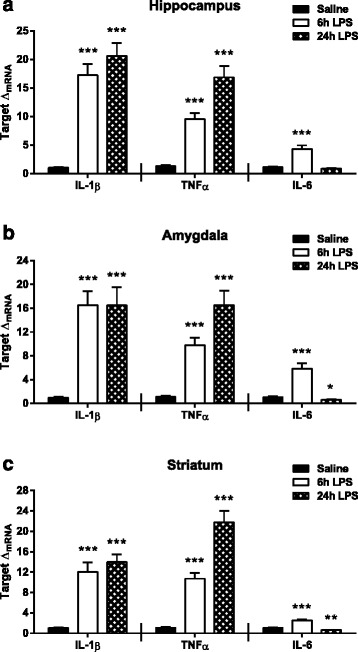
Central pro-inflammatory cytokines are elevated following peripheral LPS injections. mRNA fold changes (Δ_mRNA_) of interleukin-1β (*IL-1β*, *left panel*), tumor necrosis factor α (*TNFα*, *middle panel*), and interleukin-6 (*IL-6*, *right panel*) were measured at 6 and 24 h post LPS treatment in the (**a**) hippocampus, (**b**) amygdala, and (**c**) striatum. Data represent sample means ± SEM, *n* = 8–11 samples/group. * = post hoc comparison to saline treatment group. *p < 0.05–0.01; **p < 0.01–0.001; ***p < 0.001

Inflammation precipitates depressive-like behaviors that are controlled by neurocircuits within distinct regions of the brain, yet the impact of peripheral immune activation on pro-inflammatory cytokine expression within these regions has not been thoroughly characterized. To confirm the time course and regional response of pro-inflammatory cytokine expression following peripheral LPS challenge, interleukin-1β (IL-1β), tumor necrosis factor α (TNFα), and interleukin-6 (IL-6) were measured 6 and 24 h post treatment. In the hippocampus, IL-1β expression (Fig. 2a, left panel) was elevated relative to saline at both 6 h (*p* < 0.001) and 24 h (*p* < 0.001) post LPS injections. Similarly, TNFα expression (Fig. 2a, center panel) also increased from saline following LPS injections at both 6 h (*p* < 0.001) and 24 h (*p* < 0.001). IL-6 expression (Fig. 2a, right panel) was only elevated at 6 h (*p* < 0.001) post LPS treatment and returned to saline expression levels by 24 h. In the amygdala, both IL-1β (Fig. 2b, left panel) and TNFα expression (Fig. 2b, center panel) followed a similar pattern of increased expression at 6 h (*p* < 0.001 IL-1β; *p* < 0.001 TNFα) and 24 h (*p* < 0.001 IL-1β; *p* < 0.001 TNFα) following LPS injections. As in the hippocampus, IL-6 expression (Fig. 2b, right panel) in the amygdala was elevated at 6 h (*p* < 0.001) post LPS treatment; however, expression was significantly decreased relative to saline at 24 h (*p* = 0.018) post-LPS. IL-1β (Fig. 2c, left panel) and TNFα expression (Fig. 2c, center panel) in the striatum was elevated compared to saline at both 6 h (*p* < 0.001 IL-1β; *p* < 0.001 TNFα) and 24 h (*p* < 0.001 IL-1β; *p* < 0.001 TNFα) post LPS injections. IL-6 expression (Fig. 2c, right panel) in the striatum only increased at 6 h (*p* < 0.001) following LPS treatment and decreased relative to saline levels by 24 h (*p* = 0.001) post injections. Together, these data demonstrate that in the hippocampus, amygdala, and striatum, there is a similar pro-inflammatory cytokine temporal response following a peripheral inflammatory stimulus, such as LPS.

### Markers of glial activation increase in response to peripheral LPS challenge

**Fig. 3:**
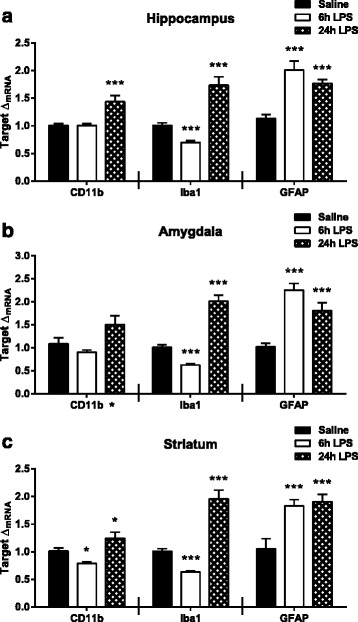
Peripheral inflammation up-regulates central expression of glial cell markers. mRNA fold changes (Δ_mRNA_) of *CD11b* (*left panel*), ionized calcium-binding adapter molecule 1 (*Iba1*, *middle panel*), and glial fibrillary acid protein (*GFAP*, *right panel*) were measured at 6 and 24 h post LPS treatment in the **a** hippocampus, **b** amygdala, and **c** striatum. Data represent sample means ± SEM, *n* = 9–11 samples/group. * = overall significance or post hoc comparison to saline treatment group. *p < 0.05–0.01; **p < 0.01–0.001; ***p < 0.001

Previous studies have demonstrated regional heterogeneity in microglia cell distribution [39] and activation following peripheral LPS injections [40]. To determine the temporal and regional expression of glial markers following peripheral LPS, mRNA expression of cluster of differentiation molecule 11b (CD11b), ionized calcium-binding adapter molecule 1 (Iba1), and glial fibrillary acidic protein (GFAP) were measured. In the hippocampus, CD11b expression (Fig. 3a, left panel), a cellular marker of microglia, was significantly elevated at 24 h (*p* < 0.001) post LPS treatment. Iba1 (Fig. 3a, center panel), another marker of microglia activation, was lower than saline at 6 h (*p* < 0.001) while expression was significantly elevated at 24 h (*p* < 0.001) post LPS injections. The astrocytic marker, GFAP (Fig. 3a, right panel), was also up-regulated relative to saline at both 6 h (*p* < 0.001) and 24 h (*p* < 0.001) following peripheral LPS treatment. In the amygdala (Fig. 3b, left, center, right panels), the expression pattern of glial markers was nearly identical to that observed in the hippocampus, with the exception that post hoc analysis revealed that CD11b expression was not significantly different from saline at either 6 or 24 h post LPS. In the striatum, CD11b expression (Fig. 3c, left panel) decreased at 6 h (*p* = 0.021) and increased at 24 h (*p* = 0.043) following LPS injections. Iba1 expression (Fig. 3c, center panel) in the striatum decreased at 6 h (*p* < 0.001) and was elevated at 24 h (*p* < 0.001) post LPS treatment. Finally, GFAP expression (Fig. 3c, right panel) was up-regulated relative to saline expression at both 6 h (*p* < 0.001) and 24 h (*p* < 0.001) after peripheral LPS. Similar to pro-inflammatory cytokines, these data suggest that glial activation is relatively uniform across discrete brain regions with up-regulation of astrocytic markers preceding microglial markers.

### Peripheral LPS challenge increases central indoleamine 2,3-dioxygenase expression

**Fig. 4:**
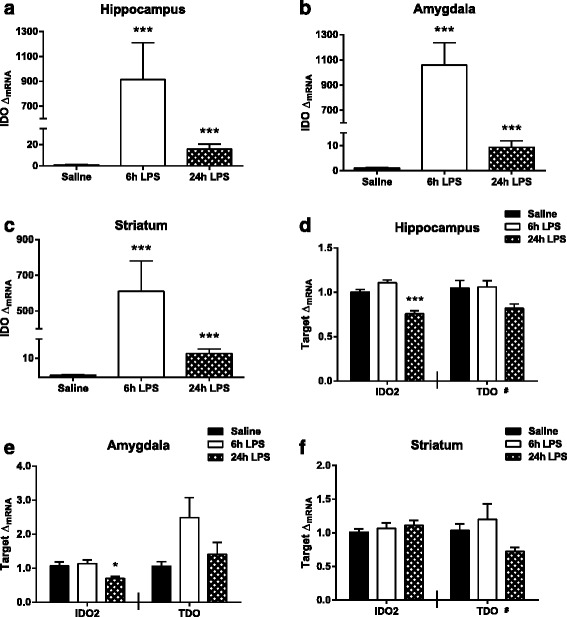
Peripheral LPS specifically increases expression of kynurenine producing enzyme, indoleamine 2,3-dioxygenase (*IDO*). mRNA fold changes (Δ_mRNA_) of IDO were assessed at 6 and 24 h following LPS injections in the (**a**) hippocampus, (**b**) amygdala, and (**c**) striatum. mRNA expression of IDO2 (*left panel*) and tryptophan 2,3-dioygenase (*TDO*, *right panel*) were also measured at 6 and 24 h post LPS in the (**d**) hippocampus, (**e**) amygdala, and (**f**) striatum. Data represent sample means ± SEM, *n* = 8–11 samples/group. ^#^,* = overall significance or post hoc comparison to saline treatment group. ^#^ *p* < 0.1–0.05; *p < 0.05–0.01; **p < 0.01–0.001; ***p < 0.001

Previous studies have established that pro-inflammatory cytokines up-regulate the expression of IDO which is necessary for the development of inflammation-associated depressive-like behaviors [19]. However, IDO2 and TDO are also expressed in the brain and whether they contribute to increased kynurenine metabolism after peripheral LPS challenge is not known. Therefore, the expression of IDO, TDO, and IDO2 (Fig. 1) was assessed. IDO expression (Fig. 4a–c) was elevated relative to saline at both 6 h (*p* < 0.001 hippocampus, *p* < 0.001 amygdala, *p* < 0.001 striatum) and 24 h (*p* < 0.001 hippocampus, *p* < 0.001 amygdala, *p* < 0.001 striatum) post LPS treatment. In the hippocampus (Fig. 4d, left panel) and amygdala (Fig. 4e, left panel), IDO-like enzyme (IDO2) expression was decreased from saline expression at 24 h (*p* < 0.001 hippocampus, *p* = 0.042 amygdala) following LPS administration, while no significant difference was observed in the striatum. There was a trend for an overall effect of time for TDO expression (*F*_2,24_ = 3.12, *p* = 0.063) in the hippocampus (Fig. 4d, right panel). In the amygdala, there was not a significant change in TDO expression (Fig. 4e, right panel) following LPS treatment. Similar to the hippocampus, striatal TDO expression (Fig. 4e, right panel) tended toward an overall effect of time (*F*_2,27_ = 2.95, *p* = 0.069). Together, these data demonstrate that IDO is the only rate-limiting enzyme of the kynurenine pathway that is significantly up-regulated following peripheral immune challenge with LPS.

### Kynurenine aminotransferases I and II are impacted in a regionally dependent manner following peripheral lipopolysaccharide

**Fig. 5:**
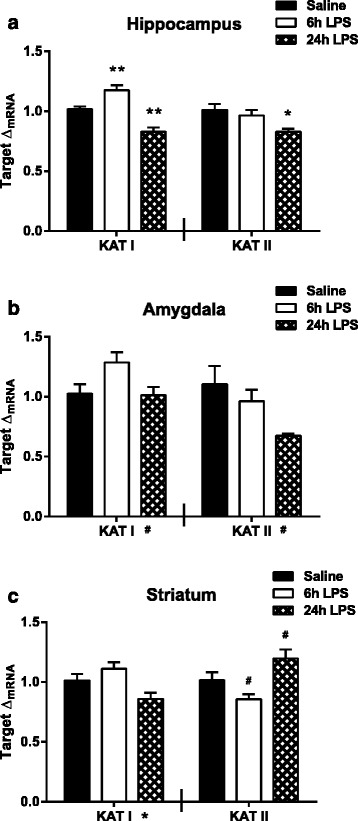
Kynurenine aminotransferases expression following peripheral LPS treatment. mRNA fold changes (Δ_mRNA_) of kynurenine aminotransferase I (*KAT I*, *left panel*) and kynurenine aminotransferase II (*KAT II*, *right panel*) were measured at 6 and 24 h post LPS in the (**a**) hippocampus, (**b**) amygdala, and (**c**) striatum. Data represent sample means ± SEM, *n* = 8–11 samples/group. ^#,^* = overall significance or post hoc comparison to saline treatment group. ^#^ *p* < 0.1–0.05; *p < 0.05–0.01; **p < 0.01–0.001; ***p < 0.001

Under basal conditions, the majority of kynurenine is metabolized to KA by kynurenine aminotransferases (KAT) I and II (Fig. 1), the expression of which is not thought to be impacted by inflammation [41]. The regional and temporal mRNA expression pattern of KATI and KATII was measured. In the hippocampus, KATI expression (Fig. 5a, left panel) was significantly elevated at 6 h (*p* = 0.005) and significantly decreased at 24 h (*p* = 0.001) relative to saline following LPS treatment. KATII expression in the hippocampus (Fig. 5a, right panel) was significantly decreased at 24 h (*p* = 0.013) following treatment with LPS. In the amygdala, both KATI (Fig. 5b, left panel, *F*_2,27_ = 3.35, *p* = 0.050) and KATII expression (Fig. 5b, right panel, *F*_2,27_ = 2.93, *p* = 0.071) trended toward significance for an overall effect of time following LPS injections. In the striatum, there was a significant overall effect of time for KATI expression (Fig. 5c, left panel, *F*_2,26_ = 5.32, *p* = 0.012) after LPS treatment, though neither 6-h expression nor 24-h expression was significantly different from saline. Similarly, striatal KATII expression (Fig. 5b, right panel, *F*_2,24_ = 7.78, *p* = 0.002) was significantly impacted by LPS in which 6-h expression (*p* = 0.096) trended toward a decrease while 24-h expression (*p* = 0.074) trended toward an increase relative to saline expression. These data suggest that peripheral LPS treatment does cause a modest time- and region-dependent change in the expression of KATI and KATII within the brain.

### Peripheral LPS induces expression changes in neurotoxic kynurenine pathway enzymes

**Fig. 6:**
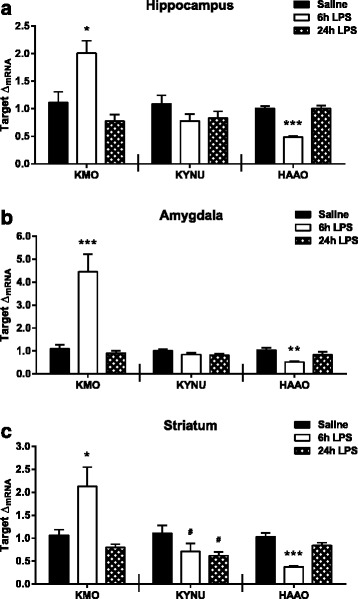
Peripheral inflammation alters expression of downstream kynurenine pathway enzymes. mRNA fold changes (Δ_mRNA_) of kynurenine 3-monooxygenase (*KMO*, *left panel*), kynureninase (*KYNU*, *middle panel*), and 3-hydroxyanthranilic acid dioxygenase (*HAAO*, *right panel*) were measured at 6 and 24 h following LPS injections in the (**a**) hippocampus, (**b**) amygdala, and (**c**) striatum. Data represent sample means ± SEM, *n* = 9–11 samples/group. ^#,^* = post hoc comparison to saline treatment group. ^#^ *p* < 0.1–0.05; *p < 0.05–0.01; **p < 0.01–0.001; ***p < 0.001

Though it has been demonstrated that peripheral LPS increases whole brain metabolism of kynurenine through the neurotoxic branch [23], the regional or temporal expression pattern of the associated pathway enzymes has not been reported. Kynurenine 3-monooxygenase (KMO), kynureninase (KYNU), and 3-hydroxyanthranilic acid dioxygenase (HAAO) (Fig. 1) mRNA was measured by real-time RT-PCR. In the hippocampus, KMO expression (Fig. 6a, left panel) was significantly elevated at 6 h (*p* = 0.024) following LPS treatment. KYNU expression (Fig. 6a, middle panel) was unchanged in response to peripheral LPS. Hippocampal HAAO expression (Fig. 6a, right panel) significantly decreased at 6 h post LPS treatment (*p* < 0.001) and returned to saline expression levels by 24 h. The same expression pattern was measured in the amygdala for each target. KMO mRNA was significantly increased (Fig. 6b, left panel, *p* < 0.001), KYNU was unchanged (Fig. 6b, middle panel), and HAAO expression (Fig. 6b, right panel) was decreased at 6 h (*p* = 0.004) but not 24 h following LPS treatment. Finally, in the striatum, KMO expression (Fig. 6c, left panel) was significantly elevated at 6 h (*p* = 0.015) and returned to saline expression at 24 h post LPS. A significant overall effect of time on striatal KYNU expression (Fig. 6c, middle panel) was apparent following LPS (*F*_2,25_ = 3.44, *p* = 0.048); however, post hoc testing revealed that 6-h expression (*p* = 0.087) and 24-h expression (*p* = 0.074) only trended to decrease from saline expression. HAAO expression (Fig. 6c, right panel) also decreased at 6 h (*p* < 0.001) and returned to saline levels 24 h following LPS treatment. Taken together, these data indicate that peripheral immune challenge with LPS causes a robust increase in the expression of KMO mRNA with a concomitant time and region-dependent down-regulation of KYNU and HAAO.

### Peripheral LPS causes a neurotoxic shift in hippocampal kynurenine metabolism

**Fig. 7:**
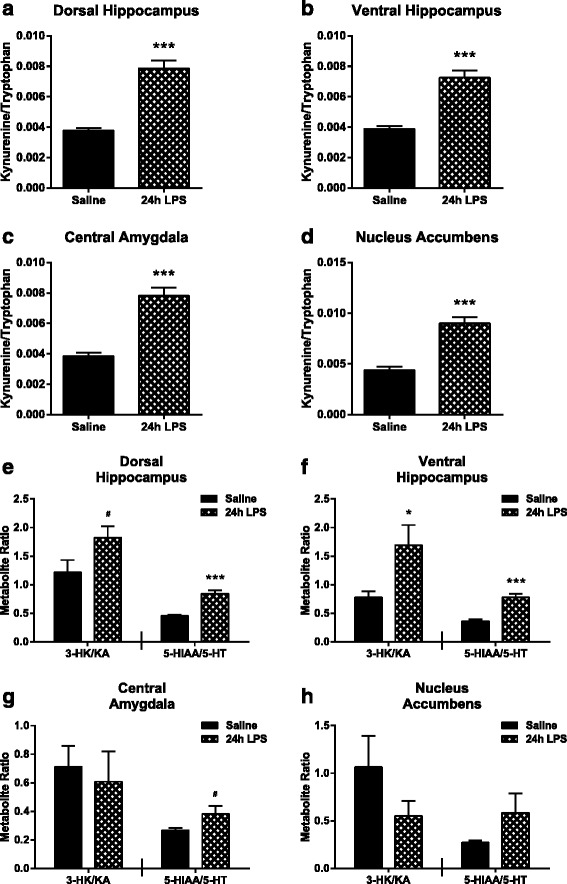
Kynurenine/tryptophan ratio and hippocampal neurotoxic kynurenine metabolite ratio are up-regulated in response to peripheral LPS. The kynurenine/tryptophan ratio was measured at 24 h following LPS injections in the (**a**) dorsal hippocampus, (**b**) ventral hippocampus, (**c**) central amygdala, and (**d**) nucleus accumbens. Metabolite ratios of 3-hydroxykynurenine/kynurenic acid (*3-HK/KA*, neurotoxic ratio, *left panel*) and 5-hydroxyindoleacetic acid/serotonin (*5-HIAA/5-HT*, serotonin turnover, *right panel*) were measured 24 h post LPS in the (**e**) dorsal hippocampus, (**f**) ventral hippocampus, (**g**) central amygdala, and (**h**) nucleus accumbens. Data represent sample means ± SEM, *n* = 7–12 samples/group. ^#^ *p* < 0.1–0.05; *p < 0.05–0.01; **p < 0.01–0.001; ***p < 0.001

Multiple studies have described the impact of LPS on central tryptophan metabolism [19, 23, 42, 43], but little is known about region-dependent tryptophan metabolism during peripheral inflammation. The kynurenine/tryptophan ratio, 3-HK/KA ratio, and 5-HIAA/5-HT ratio were determined in four brain regions 24 h post LPS treatment. LPS induced a significant increase in the kynurenine/tryptophan ratio in each brain region: dorsal hippocampus (Fig. 7a, *p* < 0.001), ventral hippocampus (Fig. 7b, *p* < 0.001), central amygdala (Fig. 7c, *p* < 0.001), and nucleus accumbens (Fig. 7d, *p* < 0.001). The balance of downstream kynurenine metabolism was shifted toward the neurotoxic branch in the hippocampus with the 3-HK/KA ratio significantly increased (dorsal; Fig. 7e, left panel, *p* = 0.054 and ventral; Fig. 7f, left panel, *p* = 0.032). The 3HK/KA ratio was not different 24 h post LPS in central amygdala (Fig. 7g, left panel) or nucleus accumbens (Fig. 7h, left panel). While serotonin levels were unchanged (data not shown), 5-HT metabolism to 5-HIAA was increased 24 h post LPS in the dorsal hippocampus (Fig. 7e, right panel, *p* < 0.001), ventral hippocampus (Fig. 7f, right panel, *p* < 0.001), and central amygdala (Fig. 7g, right panel, *p* = 0.071). While the nucleus accumbens 5-HIAA/5-HT ratio was not significantly increased (Fig. 7h, right panel). Together, these data demonstrate that LPS causes a general brain region-independent increase in tryptophan metabolism and serotonin turnover, but downstream neuroactive kynurenine metabolism occurs in a region-dependent manner.

### Lipopolysaccharide increases peripheral kynurenine metabolism toward the neurotoxic route

Because peripheral circulation is a significant source of kynurenine for the brain [37], plasma tryptophan metabolism was measured (Table 1). LPS treatment significantly increased the concentration of tryptophan (Table 1, *p* = 0.006), kynurenine (Table 1, *p* < 0.001), and 3-HK (Table 1, *p* < 0.001). The concentration of 3-HAA (Table 1), QA (Table 1), and KA (Table 1) remained unchanged 24 h after LPS treatment. The kynurenine/tryptophan ratio (Table 1, *p* < 0.001) and the 3-HK/KA ratio (Table 1, *p* < 0.001) were also significantly increased following LPS injections. These data indicate that LPS precipitates a robust up-regulation of kynurenine metabolism that is shifted toward the neurotoxic branch; however, neuroactive metabolic end products were not increased.

**Table 1:** Peripheral inflammation increases flux through the kynurenine pathway of tryptophan metabolism

Metabolite	Concentration (μM)		*t* test
	Saline	24 h LPS	*p* value
Tryptophan	94.93 (7.1)	119.53 (3.9)	*p* < 0.01
Kynurenine	0.83 (0.08)	2.40 (0.2)	*p* < 0.001
3-Hydroxykynurenine (3-HK)	0.16 (0.02)	0.27 (0.04)	*p* < 0.001
3-Hydroxyanthranilic acid (3-HAA)	3.99 (0.3)	4.87 (0.4)	n.s.
Quinolinic acid (QA)	0.98 (0.06)	0.97 (0.07)	n.s.
Kynurenic acid (KA)	0.24 (0.03)	0.27 (0.01)	n.s.
Kynurenine/tryptophan ratio	0.0092 (0.0004)	0.020 (0.005)	*p* < 0.001
3-HK/KA ratio	0.55 (0.04)	1.08 (0.07)	*p* < 0.001

Kynurenine pathway metabolites (μM) were assessed by LC/MS in the plasma 24 h following saline or LPS treatment. Metabolites measured include tryptophan, kynurenine, 3-hydroxykynurenine (3-HK), 3-hydroxyanthranilic acid (3-HAA), quinolinic acid (QA), and kynurenic acid (KA). Kynurenine/tryptophan and 3-HK/KA ratios were calculated. Data represent sample means (SEM) and were analyzed using a *t* test (results denoted in table). *n* = 7–12 samples/group, *n.s.* not significant

## Discussion

Under the guidelines of the RDoC framework, a renewed emphasis has been placed on understanding regionally distinct structural, cellular, and molecular events involved in the pathogenesis of neuropsychiatric disease [29, 30]. Therefore, the objective of this study was to characterize the LPS-induced neuroinflammatory response in brain regions relevant to depression-related symptoms as previous literature has described these data only at the whole brain level. Up-regulation of pro-inflammatory cytokine expression (IL-1β, TNFα, and IL-6) followed the same temporal pattern in each of the three regions studied (hippocampus, amygdala, and striatum) following peripheral LPS challenge. The relative magnitude of the LPS-induced cytokine response was similar as well. Likewise, glial cell markers (CD11b, Iba1, and GFAP), in general, were elevated in a region-independent manner. Interestingly, up-regulation of astrocytic marker GFAP preceded the subsequent increase in microglial markers CD11b and Iba1. Consistent with studies demonstrating that IDO expression is induced by pro-inflammatory cytokines, IDO expression was robustly up-regulated in a region-independent manner, peaking at 6 h following LPS treatment, while IDO2 and TDO remained largely unchanged. Interestingly, the expression of neurotoxic kynurenine pathway enzymes downstream of IDO (KMO, KYNU, and HAAO) was also similar between brain regions; however, each enzyme had a unique response pattern following peripheral inflammation. KMO expression increased, KYNU expression remained unaffected, and HAAO expression decreased as an immediate response to LPS treatment. On the opposing metabolic branch, KAT enzymes (I and II) were significantly down-regulated in the hippocampus, 24 h after LPS treatment. In accord with the mRNA data, functional assessment of kynurenine metabolism showed that the kynurenine/tryptophan ratio increased approximately twofold following peripheral immune activation in the same pattern throughout the regions assessed. Interestingly, the 3-HK/KA ratio, which reflects the relative neurotoxic/neuroprotective metabolic balance, was only up-regulated in the hippocampal regions (dorsal and ventral) following LPS treatment. Together, these data demonstrate that peripheral immune challenge results in region-independent induction of neuroinflammation and kynurenine production, while downstream metabolism of kynurenine is region specific.

To characterize the neuroinflammatory response associated with peripheral immune activation, pro-inflammatory cytokine and glial cellular marker expressions were assessed. The expression of all three pro-inflammatory cytokines, IL-1β, TNFα, and IL-6 were increased 6 h after LPS injections in all three brain regions, similar to previously published data from the hippocampus, cortex, and hypothalamus [17, 44]. This early central pro-inflammatory response drives the development of LPS-induced sickness behavior (lethargy, decreased appetite, reduced social interactions, fever) [45, 46]. At 24 h post-LPS treatment, IL-1β and TNFα expression remained elevated while IL-6 returned to saline expression levels, also an effect similar to previously published results [17]. It is important to note that under the experimental conditions of this study, 24 h after LPS treatment corresponds to when depressive-like behaviors have been assessed previously [47]. It is possible that the culmination of IL-6 prior to IL-1β and TNFα expression is associated with the resolution of a specific behavior, such as fever [48], while other behaviors persist at 24 h post-LPS and IL-1β and TNFα play an important role in the development of those behaviors. It is important to note that using steady-state mRNA analysis of brain neuroinflammatory targets has both merit and limitations. In our study, mRNA targets allow for an extremely sensitive and quantitative measure of neuroinflammatory gene expression. Since many pro-inflammatory cytokines, including IL-1β, TNFα, and IL-6, are able to enter the brain via transport across the blood brain barrier [49, 50], determination of mRNA expression ensures that any changes being measured reflect the local brain expression of the targets, not the appearance/accumulation of peripherally secreted cytokines. However, it is possible that variability in translation dynamics of mRNA to protein could influence the expression of *de novo* synthesized neuroinflammatory targets.

Interestingly, the early up-regulation of central pro-inflammatory cytokine mRNA was coupled with a general down-regulation of microglial markers (CD11b and Iba1) and increase of astrocytic GFAP. At both time points after LPS, expression of astrocyte cell marker GFAP was up-regulated, which is consistent with a previous study demonstrating that central LPS administration increases GFAP expression [51]. Microglia are known to be the predominant cellular producers of pro-inflammatory cytokines in the brain, and Iba1 expression and/or staining are often reported as a proxy of activation state [52]. The incongruence in the expression patterns between the gene targets in our study suggests that expression of pro-inflammatory cytokines precede the up-regulation of microglia-specific markers. Additionally, utilization of CD11b or Iba1, in the absence of morphologic or other complementary data, appears to be an insufficient representation of microglia activation state. Moreover, the temporal difference between up-regulation of astrocyte versus microglial markers suggests the possibility of a dynamic role for these two cell types in mediating the neuroinflammatory response. This could be particularly relevant in terms of kynurenine metabolism which is a pathway compartmentalized between astrocytes and microglia in the brain [24, 53]. It is interesting to note that both CD11b and GFAP are increased in their respective cell populations by increases in nitric oxide (NO) production [54, 55], which can be generated by elevations in pro-inflammatory cytokines and kynurenine metabolite QA [56–58]. Further investigation is warranted to more precisely explore this phenomenon.

The expression of all known rate-limiting kynurenine producing enzymes, IDO, IDO2, and TDO, was assessed to better understand how tryptophan is metabolized following peripheral LPS. IDO expression was increased at both 6 and 24 h post LPS injections in each brain region assessed, consistent with two previously published studies [17, 44]. IDO2 is a more recently discovered homologue of IDO with much lower enzymatic activity compared to IDO [59]. In the hippocampus and amygdala, IDO2 expression decreased at 24 h following LPS while otherwise remaining unchanged, which follows conflicting previous studies demonstrating that pro-inflammatory stimuli increased or had no impact on IDO2 mRNA [60, 61]. Overall, TDO expression did not increase in any brain region at either time point in response to LPS treatment, confirming previous reports demonstrating that TDO expression is up-regulated by glucocorticoids [62]. Together, these data confirm that IDO is the only rate-limiting kynurenine pathway enzyme up-regulated in response to peripheral LPS, demonstrating increased capacity for *de novo* kynurenine production during neuroinflammatory conditions.

The metabolism of kynurenine is physically compartmentalized within the brain [24, 53]. Astrocytes express mainly KATs (not KMO) for conversion of kynurenine to KA [63, 64]. Microglia express KMO (not KATs) that metabolizes kynurenine along the neurotoxic branch, leading to the potential formation of several neurotoxic metabolites [65, 66]. Contemporary research suggests that the relative balance of these opposing metabolic branches, rather than simply changes in the levels of individual metabolites, constitutes the pathogenic potential of kynurenine metabolism [53]. Though LPS treatment increased GFAP expression and presumably astrocytic activation, there was only a significant increase in KATI expression in the hippocampus at 6 h post injections. Further, at 24 h post-LPS, KATI and KATII expression was reduced in the hippocampus. A previous expression study reported that LPS treatment did not affect KATII expression in the hippocampus and cortex [44], in contrast with our results in the hippocampus. The data reported here, suggesting that KAT expression (I and II) is generally unchanged by LPS treatment, are consistent with previous findings that demonstrate that whole brain KA concentration levels do not increase at 24 h post-LPS treatment [23]. Instead, that same study demonstrated that peripheral LPS-induced elevations in central kynurenine result in increases in 3-HK, 3-HAA, and QA on the neurotoxic branch, mediated through KMO in microglia [23]. Interestingly, KMO was the only neurotoxic enzyme up-regulated following LPS treatment, while KYNU expression did not change and HAAO decreased expression following LPS treatment. In a previous study, at 4 h post-LPS, KMO expression decreased in the cortex, and at 24 h post-LPS, expression increased in both the cortex and hippocampus [44]. The experimental conditions, such as species (rats) and LPS dose (0.25 mg/kg), are likely contributors to the differences in expression kinetics between the two studies. In our study, it is noteworthy to consider the possibility that HAAO expression is down-regulated as a compensatory response to the increased expression of KMO and the potential increase in flux of metabolites through the neurotoxic branch. Interestingly, the disconnect between expression of microglia cell markers (Iba1, CD11b) and KMO provides further evidence that the functional cellular response is not necessarily accurately reflected by measuring standard microglial markers.

To further explore the effects of LPS on kynurenine metabolic balance within behaviorally relevant brain regions, we calculated ratios that (1) reflected IDO activity and up-regulation of overall kynurenine metabolism (kynurenine/tryptophan), (2) indicated the relative balance of kynurenine metabolism toward the neurotoxic branch (3-HK/KA), and (3) assessed serotonin turnover (5-HIAA/5-HT). Twenty-four hours post LPS was chosen as the sampling time point based on established behavioral kinetics [47]. Additionally, mRNA increases measured at 6 h post-LPS require time in order for protein translation to begin impacting metabolism relevant to behavior changes. In all of the regions assessed, the kynurenine/tryptophan ratio was elevated as predicted based on IDO expression data and previously published data [18, 19, 23, 67]. However, assessment of the 3-HK/KA ratio, indicative of metabolism down the neurotoxic kynurenine branch, revealed that peripheral LPS treatment only increased this neurotoxic ratio in the dorsal and ventral hippocampus. Relevant to this, a recent study suggested that microglia in separate regions of the brain have functionally distinct characteristics, specifically that those in the hippocampus are more pro-inflammatory in nature [68]. As the main producers of neurotoxic kynurenine metabolites, these more immunovigilant hippocampal microglia might drive the region-specific shift toward neurotoxic metabolism observed here. Alternatively, it is possible that astrocytic production of KA may follow a region-dependent pattern, although this has yet to be directly investigated. Further, while not significant, increasing statistical power may actually reveal that the 3-HK/KA ratio decreases in the nucleus accumbens in response to peripheral LPS treatment. This will require additional future experiments. Interestingly, recent data collected from a chronic social defeat model, also associated with a peripheral immune response, demonstrated regional variation in the up-regulation of central kynurenine and 3-HK production [69]. However, when brain region kynurenine metabolites were assessed in response to peripheral CD40 antibody treatment, which induces inflammation, both kynurenine and 3-HK were elevated in all of the regions assessed [70]. Previous studies have demonstrated that peripheral LPS treatment increases serotonin turnover in the whole brain [19]. In line with those data, the 5-HIAA/5-HT ratio was increased or trended to increase in all the regions assessed in this study, except the nucleus accumbens. Together, these data illustrate, for the first time, that brain kynurenine metabolism is regulated in a regionally distinct manner following peripheral immune challenge with LPS. Our recently published data support the notion that this neurotoxic shift is functionally relevant, as targeted deletion of the KMO gene protects mice from LPS-induced disruption in a hippocampal-dependent behavioral task [26].

As a control measure, tryptophan and kynurenine metabolites were assessed in the plasma 24 h after LPS treatment (Table 1). As predicted, LPS increased kynurenine, 3-HK, the kynurenine/tryptophan ratio, and the 3-HK/KA ratio and did not change KA. Curiously, the tryptophan concentration increased as well, contrary to previous clinical and preclinical studies demonstrating that inflammation is associated with a decrease in tryptophan or ‘tryptophan depletion’ [16, 19, 71]. Further, there were no changes in 3-HAA or QA after LPS treatment, again differing from previous studies showing LPS elevates peripheral and central QA as well as central 3-HAA [23, 71]. The current study is the first to explore kynurenine metabolic balance downstream of IDO in C57BL/6J inbred mice while previous studies were carried out in CD-1 outbred mice [23]. The dissociation between central and peripheral downstream kynurenine metabolites has also been observed in the clinic, when cerebrospinal fluid QA was elevated without an increase in plasma QA in hepatitis C patients receiving interferon-α immunotherapy [72]. Importantly, our data revealed that even though kynurenine/tryptophan doubles in both the peripheral circulation and in each of the brain regions measured (supporting the notion of the periphery as a primary source of these amino acids), the manner in which kynurenine metabolism proceeds is not simply a function of substrate levels. The distinct 3-HK/KA ratios, in the face of uniform increases in kynurenine/tryptophan ratios, further underscore that downstream brain region metabolism is regionally discrete and locally regulated. Understanding how local metabolic changes impact the behaviors mediated by distinct brain regions remains an important area for future study. Together, these data demonstrate that peripheral LPS up-regulates the metabolism of tryptophan and kynurenine to 3-HK, in both the periphery and the brain. However, region-dependent regulation of kynurenine metabolic balance within the brain is potentially pertinent to the pathogenesis of depression and is a therapeutically relevant finding.

## Conclusions

The main goal of this study was to provide a better understanding of the regional neuroinflammatory response following peripheral treatment with LPS. This response involved mRNA expression (Fig. 1) of pro-inflammatory cytokines (IL-1β, TNFα, IL-6), glia cell markers (CD11b, Iba1, GFAP), and kynurenine pathway enzymes (IDO, IDO2, TDO, KATI, KATII, KMO, KYNU, HAAO) at 6 and 24 h post LPS in relevant brain regions (hippocampus, amygdala, striatum). Additionally, kynurenine metabolite ratios (kynurenine/tryptophan, 3-HK/KA, 5-HIAA/5-HT) were calculated from previously collected data at 24 h post-LPS in relevant brain regions (dorsal and ventral hippocampus, central amygdala, nucleus accumbens). In general, these data demonstrated that in response to peripheral LPS treatment, pro-inflammatory cytokines and glia cell makers increase similarly throughout the brain. Kynurenine pathway enzymes have distinct temporal expression changes following LPS, which are region-independent; however, the metabolic balance is regulated in a region-dependent manner. Further analysis of the individual metabolite concentrations and additional brain regions will advance the understanding and potential implications of brain region-specific kynurenine metabolism. The possibility that individual depression-related behaviors are driven by regionally defined disruptions in kynurenine metabolism provides a specific target for future pharmacotherapy developments.

## Abbreviations

3-HAA, 3-hyroxyanthranilic acid; 3-HK, 3-hydroxykynurenine; 5-HIAA, 5-hyrdoxyindoleacetic acid; 5-HT, serotonin; CD11b, cluster of differentiation molecule 11b; CSF, cerebrospinal fluid; fMRI, functional magnetic resonance imaging; GFAP, glial fibrillary acidic protein; HAAO, 3-hyroxyanthranilic acid dioxygenase; HPA, hypothalamic-pituitary-adrenal; IDO, indoleamine 2,3-dioxygenase; i.p., intraperitoneal; Iba1, ionized calcium-binding adapter molecule 1; IFN-α, interferon-α; IL-1β, interleukin-1β; IL-6, interleukin-6; KA, kynurenic acid; KAT, kynurenine aminotransferase; KMO, kynurenine 3-monooxygenase; KYNU, kynureninase; LC/MS, liquid chromatography/mass spectrometry; LPS, lipopolysaccharide; MADRS, Montgomery-Asberg Depression Rating Scale; NMDAR, *N*-methyl-d-aspartate receptor; NO, nitric oxide; QA, quinolinic acid; RDoC, Research Domain Criteria; SEM, standard error of the mean; TDO, tryptophan 2,3-dioxygenase; TNFα, tumor necrosis factor α; UTHSCSA, University of Texas Health Science Center at San Antonio; α_7_nAChR, α_7-_nicotinic acetylcholine receptor
